# The Regulatory Roles of Long Noncoding RNAs in Acute Myeloid Leukemia

**DOI:** 10.3389/fonc.2019.00570

**Published:** 2019-07-09

**Authors:** Michelle Ng, Dirk Heckl, Jan-Henning Klusmann

**Affiliations:** Department of Pediatrics I, Martin Luther University Halle-Wittenberg, Halle, Germany

**Keywords:** acute myeloid leukemia, long noncoding RNA, regulatory RNA, antisense transcripts, competing endogenous RNA, chromatin looping, lncRNA profiles

## Abstract

In this post-genomic era, long noncoding RNAs (lncRNAs) are rapidly gaining recognition for their crucial roles across diverse biological processes and contexts. The human blood system is no exception, where dozens of lncRNAs have been established as regulators of normal and/or malignant hematopoiesis, and where ongoing works continue to uncover novel lncRNA functions. Our review focuses on lncRNAs that are involved in the pathogenesis of acute myeloid leukemia (AML) and the mechanisms through which they control gene expression in this disease context. We also comment on genome-wide sequencing or profiling studies that have implicated large sets of lncRNAs in AML pathophysiology.

## Background: On lncRNAs and AML

Hematopoietic stem cell (HSC) homeostasis and lifelong blood formation rely on a complex interplay between many different pathways. These include growth factors, signaling cascades, and transcription factors, all of which interact to control the fine balance between self-renewal, quiescence, proliferation, and differentiation (1). Deregulation of this critical interplay can result in malignancy. One example is acute myeloid leukemia (AML), a heterogeneous hematologic disease characterized by the uncontrolled proliferation of undifferentiated myeloid precursors (blasts) (2). This phenotype can be driven by genetic abnormalities that enhance self-renewal and block differentiation, such as chromosomal translocations [e.g., t(15;17)[*PML-RARA*], t(9;22)[*BCR-ABL*], inv(16)[*CBFB-MYH11*], t(8;21)[*RUNX1-ETO*], *MLL* rearrangements] and gene mutations (e.g., *DNMT3A, NPM1, CEBPA, IDH1/2, TET2, FLT3*-ITD), the majority of which are well-defined (3–6). However, our current understanding of the underlying genetic basis of AML hinges on decades of protein-centered research. The contribution of noncoding regions to the initiation, maintenance, and evolution of AML remains to be fully revealed.

It is well-known that only about 2% of the human genome encodes protein, while 70–90% is transcribed, giving rise to an enormous number and variety of noncoding transcripts (7, 8). Among these, long noncoding RNAs (lncRNAs) comprise a substantial proportion (9) that are drawing significant attention as novel regulatory RNAs with roles in diverse physiological processes. LncRNAs are operationally defined as transcriptional products over 200 nucleotides (nt) long that ostensibly lack open reading frames (10, 11). Based on their location relative to coding loci along the genome, they can be further subdivided into four groups: (1) sense, (2) antisense, (3) intronic, or (4) intergenic (12, 13). Some mechanisms that have been described for lncRNAs, so far, include cooperating with gene regulatory circuits in the nucleus (e.g., transcription factor recruitment or chromatin looping), blocking translation, sequestering microRNAs (miRNAs), getting processed into small RNAs, and encoding micropeptides (14–18). Regulating chromatin configuration appears to represent a recurring mechanistic theme among newly characterized lncRNAs, but in general, lncRNA functions do not correspond with their current classification, and we cannot yet conclusively point to unifying pathways. Nonetheless, the picture is still incomplete, and when lncRNAs are better understood as a class, an improved, functionally defined classification system may emerge.

Given the recent rapid expansion of the lncRNA research field and its exposition of lncRNA involvement across a range of key biological processes, it is no surprise that lncRNAs also regulate the blood system. We now know a number of individual lncRNAs that influence lineage decisions along the hematopoietic hierarchy and whose deregulation can result in hematologic malignancy (19–29). These examples are discussed in comprehensive review articles that synthesize our present understanding of lncRNA in normal and malignant hematopoiesis (30–33). While these articles take a broad view on the intersection of hematology and lncRNA biology, this research area has grown to the point where targeted commentaries for different leukemia subtypes may prove beneficial—not least because of the context specificity of lncRNA expression, including between cancers (34, 35). Here, we provide a focused, updated review of lncRNAs that are specifically relevant to AML.

## lncRNAs Implicated in AML Pathophysiology

In recent years, several large-scale expression studies have implicated lncRNAs in the pathogenesis of AML by demonstrating the association of distinct lncRNA profiles with genetically defined subtypes of AML (27, 36–40). In the first study of this kind, Garzon et al. profiled lncRNA expression in patients over 60 years old with untreated cytogenetically normal (CN) AML. They discovered distinctive lncRNA signatures associated with major molecular subtypes of AML including *FLT3*-ITD, *NPM1, CEBPA, IDH2*, and *RUNX1* mutations. Whole-transcriptome sequencing in a cohort of younger adults (<60 years old) similarly revealed lncRNA expression profiles associated with *FLT3*-ITD, *NPM1*, and *CEBPA* mutations (38). Interestingly, lncRNAs located antisense to *HOX* genes featured among those that were upregulated in *NPM1*-mutated cases, particularly in the cohort of older adults. In both studies, the authors derived a prognostic score based on a small subset of survival-associated lncRNAs and demonstrated its robust performance as an independent predictor of clinical outcome. Papaioannou et al. additionally correlated their prognostic lncRNAs with messenger RNA (mRNA) and miRNA expression, thereby linking the lncRNAs to cancer-related pathways like leukocyte activation, inflammation, and apoptosis through guilt by association. Last, a smaller study of CN-AML patients uncovered an lncRNA signature that depended on the mutational status of *NPM1* (37). The authors identified and validated a minimal set of 12 lncRNAs that could discriminate between *NPM1*-mutated and *NPM1*-wild type cases and initiated the preliminary characterization of one lncRNA, *XLOC_109948*, which they propose is involved in drug sensitivity.

Besides recurrent mutations, distinct lncRNA profiles have also been associated with certain cytogenetic subgroups of AML (27, 39). For example, using unconventional library preparation and assembly approaches, Zhang et al. uncovered a set of four noncoding transcripts that are specifically and highly expressed in patients harboring the *PML-RARA* translocation. These included a novel, previously unannotated lncRNA, and *MEG3*, an established maternally expressed lncRNA (39). Our own group has also reported subtype-specific fingerprint lncRNAs for six major subgroups of pediatric AML [inv(16), t(8;21), t(10;11), t(9;11), acute megakaryoblastic leukemia (AMKL), and Down syndrome myeloid leukemia (ML-DS)] (27). We further compared these expression profiles with those of healthy blood populations and found HSC signatures that are upregulated in AML blasts. These lists and others, spanning across the human hematopoietic lineages and pediatric AML subtypes, are publicly available as an online resource (www.lncscape.de), which aims to provide an outline of the lncRNA landscape behind normal and malignant hematopoiesis.

The molecular and cytogenetic subtype-specific expression patterns of lncRNAs in AML samples suggest their crucial contribution to the pathophysiology of this disease. In addition, this phenomenon poises lncRNAs as attractive targets for new therapeutic approaches where the specific targeting of oncogenic proteins has been unsuccessful. However, regarding the role of subgroup-associated lncRNA profiles in leukemogenesis, it remains ambiguous whether they are significant in their own right or whether they represent passenger events driven by genetic aberrations affecting coding genes. Shedding light on this question to some extent is Mer et al.'s exploration of an lncRNA-centered stratification system for AML patients (40). Based solely on lncRNA expression, the authors distinguished four molecular subtypes that differ in prognoses and active pathways and that behave independently of the European Leukemia Net (ELN) risk classification groups. However, although these lncRNA-based subgroups lacked high concordance with conventional clinical or genetic factors, there was, nonetheless, some association with traditional molecular determinants such as mutations in *CEBPA, NPM1, TP53*, and *FLT3-ITD* (40). Another recent study of CN-AML cases with leukemia stem cell (LSC)-associated core-enriched gene expression signatures (CE-GES) discovered a set of 111 lncRNAs that strongly correlate with the LSC signature (41). One of the most upregulated LSC-associated lncRNAs, *DANCR*, was confirmed in functionally validated LSC populations, and its knockdown was shown to reduce self-renewal capacity. The authors further demonstrated that targeting *Dancr in vivo* in a murine model of AML prolonged the survival of mice after secondary transplantation (41).

All of the above studies implicate lncRNAs in the pathogenesis of AML, and De Clara et al. and Bill et al. additionally indicated their functional relevance and therapeutic potential. Other publications featuring lncRNA-based prognostic scoring systems are also beginning to emerge (42–45). Even so, extensive functional work is required to delineate the interactions between lncRNAs and known driver events and to better understand how each factor can influence the development and progression of AML. With lncRNA research continuing to grow at such a rapid rate, it will soon become crucial that we integrate this body of knowledge into our disease models and treatment practices toward ultimately improving clinical outcomes for patients.

## Mechanisms of lncRNA-Mediated Gene Regulation in AML

Starting only a few years ago, the field of lncRNA research underwent—and is continuing to undergo—an exponential expansion. Dozens of articles are published per month describing lncRNA functions in a wide variety of healthy and diseased cell contexts. While this is very exciting for those of us who dream of someday being able to look up lncRNA functions as easily as we currently do for proteins, the field is still young, and in-depth mechanistic investigation is missing for many newly “characterized” lncRNAs. Further complicating the situation, some lncRNAs may produce different outcomes on gene regulation in different cell contexts or act *via* multiple pathways in a single context. Given the current, incomplete state of the literature—where, in many cases, one lncRNA is reported in a range of cell types along with an equal range of dissimilar mechanisms—it is difficult to distinguish between these two possibilities. By extension, it is also challenging to group lncRNAs based on their mechanisms. Nonetheless, we compiled a catalog of lncRNAs that have specifically been shown to play regulatory roles in AML cells (Table 1). While the list is not exhaustive, it summarizes the best examples of lncRNAs in AML pathogenesis and their mechanisms of action, which are discussed in detail in the rest of this review.

**Table 1:** A summary of lncRNAs that regulate gene expression in AML and their roles in leukemia cells.

**LncRNA**	**Putative role**	**Function in AML cells**	**Mechanism of action**	**References**
*HOTAIRM1*	Tumor suppressor	Regulates myeloid maturation, cell cycle, and autophagy	Activates proximal *HOXA* and *CD11b*/*CD18*/CD11c expression and represses CD49d; sponges miR-20a/miR-106b/miR-125b	(46–49)
*HOXA-AS2*	Oncogenic	Mediates resistance to apoptosis and Adriamycin	Sponges miR-520c-3p, thereby increasing S100A4	(50, 51)
*HOTAIR*	Oncogenic	Promotes leukemic phenotypes by modulating *c-KIT* and *p15*	Sponges miR-193a away from *c-KIT* and epigenetically silences *p15*, perhaps *via* PRC2 or LSD1^?^	(52, 53)
*CCAT1*	Oncogenic	Inhibits myeloid maturation and promotes proliferation	Sponges miR-155 away from *c-MYC*	(54)
*UCA1*	Oncogenic	Promotes chemoresistance, glycolysis, and proliferation; activated by CEBPα-p30	Sponges miR-125a away from *HK2*, and miR-126 from *RAC1*; translationally represses p27^!^	(55–57)
*PU.1-AS*	Oncogenic^*^	Negatively regulates *PU.1* expression	Interferes with *PU.1* translation by binding eIF4A	(58)
*IRAIN*	Tumor suppressor^°^	Regulates expression of *IGF1R in cis*	Mediates enhancer looping to *IGF1R* promoter	(59)
*PVT1*	Oncogenic	Regulates expression of *MYC in cis*; promotes proliferation and survival	Alternate TSS are enhancers that loop to *MYC*^!^; promoter acts as a DNA boundary element^?^	(60–62)
*RUNXOR*	Oncogenic^°^	Upregulated in AML blasts and after Ara-C treatment^!^; likely regulates *RUNX1*	Interacts with chromatin to form intra/interchromosomal loops, and with RUNX1 and EZH2	(63)
*GAS6-AS2*	Oncogenic	Mediates resistance to cytarabine (Ara-C)	Positively regulates *GAS1* and *AXL* expression in the latter case *via* promoter methylation by DNMT^!^	(64)
*TUG1*	Oncogenic	Confers Adriamycin resistance	Epigenetically silences *miR-34a via* EZH2-dependent deposition of H3K27me3 at the promoter	(65)
*MIR100HG/MONC*	Oncogenic	Required for maintenance and self-renewal of AMKL	Enhances erythroid progenitors	(66)
*LINC-223*	Tumor suppressor	Promotes monocytic differentiation	Acts as a decoy against oncogenic miR-125 family members	(67)
*ANRIL*	Oncogenic	Maintains AML cell survival/proliferation and regulates glucose metabolism	Activates ADIPOR1/AMPK/SIRT1 expression	(68)
*NEAT1*	Tumor suppressor	Decreases proliferation and increases apoptosis	May act as a ceRNA on the miR-23a-3p/*SMC1A* axis	(69)
*H19*	Oncogenic	Behaves like an oncogenic lncRNA; may be involved in telomerase activity	May act as a ceRNA on miR-19/*ID2*; mediates hTERT2/*hTR* interaction	(70–72)
*CASC15*	Oncogenic^*^	Promotes myeloid over B-cell development^≠^	May positively regulate *SOX4* transcription *via* YY1^?^	(73)
*CCDC26*	Tumor suppressor^*^	May regulate cellular response to starvation!	May negatively regulate c-KIT!	(74)
*MEG3*	Tumor suppressor	Acts as a tumor suppressor, is regulated by WT1/TET2	Activates *p53*, downregulates DNMT3A through MDM2/RB1	(75)
*WT1-AS*	Tumor suppressor^*^	Mediates *WT1* induction under hypoxic conditions	Regulates *WT1* expression *in cis*	(76)

*The order corresponds to their sequence of appearance in the manuscript. The lncRNAs are grouped according to their mechanisms of action, with different groups separated by double lines in the table*.

? *Mechanism described in other tumor contexts; remains to be confirmed in AML*.

! Mechanism described in K562 cells (chronic myeloid leukemia, CML); implicated in AML.

* Speculation based on the regulation of coding genes; cellular phenotype not yet characterized.

° Speculation based on expression levels in patient samples; cellular phenotype not yet characterized.

≠ *Experiment conducted in the murine system; remains to be validated in human cells*.

## Competing Endogenous RNAs

**Figure 1:**
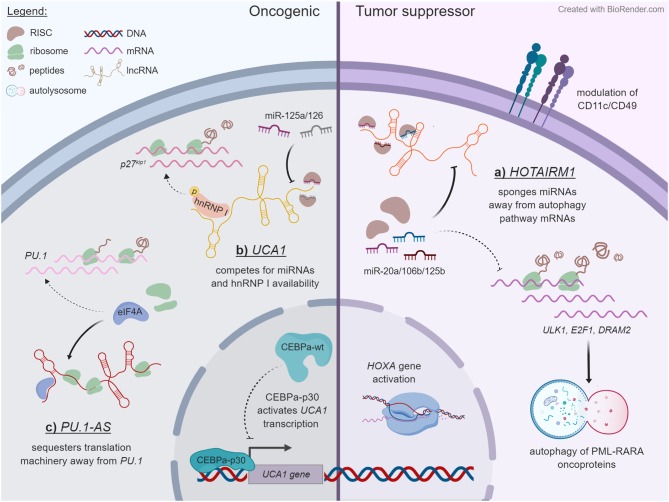
Some examples of lncRNA mechanisms that unfold in the cytoplasmic compartment. Dashed lines specify pathways that are reduced upon lncRNA expression, whereas solid lines indicate outcomes of lncRNA expression. The examples are ordered in a counterclockwise manner. **(A)** *HOTAIRM1* acts as a decoy for miR-20a, miR-106b, and miR-125b in NB-4 cells, titrating them away from mRNAs that encode regulators of autophagy, and thereby enhancing the degradation of PML-RARA oncoprotein through this pathway. *HOTAIRM1* also modulates the expression of *HOXA* genes and of cell surface lineage markers CD11c and CD49d through an undetermined mechanism. **(B)** In contrast to wild-type CEBPα, truncated CEBPα-p30 transactivates the *UCA1* promoter in AML cells with CEBPα mutations. The *UCA1* transcript functions as a sponge on miR-125a and miR-126 and additionally competes with the *p27*^*kip*1^ mRNA for stabilizing interactions with hnRNP I. **(C)** *PU.1-AS* lncRNAs are polysome associated and interfere with *PU.1* protein expression by competitively binding the translation initiating factor eIF4A.

The deregulation of *HOX* genes has long been accepted as an important mechanism of leukemogenesis, often in connection with chromosomal translocations involving *MLL* (77). It is therefore unsurprising that lncRNAs transcribed from the *HOX* loci have also been discovered to exert regulatory roles in AML. For example, *HOTAIRM1* is an intergenic lncRNA (lincRNA) located between *HOXA1* and *HOXA2* that is transcribed antisense to the *HOXA* cluster (46), and it is one of the best-studied lncRNAs in the blood system. Under ordinary circumstances, *HOTAIRM1* expression is restricted to the myeloid lineage (46). In the NB-4 human acute promyelocytic leukemia (APL) cell line, it is strongly upregulated during all-trans retinoic acid (ATRA)-driven granulocytic differentiation, where it selectively modulates the induction of *HOXA1*/*HOXA4* and of the myeloid maturation markers *CD11b, CD18*, and *CD11c*, meanwhile repressing *CD49d* (46, 47). *HOTAIRM1* was shown to promote ATRA-driven cell cycle arrest, suggesting that *HOTAIRM1*-mediated gene expression changes may regulate a switch from a proliferative phase toward granulocytic maturation (47). An alternate, detailed mechanism put forward by Chen et al. contends that *HOTAIRM1* regulates autophagy and thus the degradation of the PML-RARA oncoprotein, which characterizes APL and drives leukemogenesis in this subtype (48). *HOTAIRM1* appears to accomplish this by acting as a competing endogenous RNA (ceRNA) and thereby sequestering miR-20a, miR-106a, and miR-125b away from their target mRNAs in the autophagy pathway—*ULK1, E2F1*, and *DRAM2* (48) (Figure 1A).

While these findings provide an elegant *trans* mechanism for *HOTAIRM1*, another study from around the same time frame proposed a *cis*-regulatory mode of action within the *HOXA* cluster. Wang et al. reported *HOTAIRM1*'s involvement in controlling the three-dimensional chromatin organization behind *HOXA* gene activation in NT2-D1 carcinoma cells (49). Interestingly, modulating *HOTAIRM1* exerted opposite effects on *HOXA4/5* expression in NB-4 and NT2-D1 cells and produced chromatin conformation changes in NT2-D1 that were absent in NB-4 cells. These findings provide experimental evidence of the often-cited tissue-specific manner of lncRNA function (7–9) and offer a reminder to exercise caution when generalizing their mechanisms across cell types and contexts. Notably, both studies identified multiple *HOTAIRM1* isoforms, which may explain the differences in *HOTAIRM1*-regulated *HOXA* gene expression between the two cell lines (48, 49). The primary *HOTAIRM1* transcript detected in ATRA-treated NB-4 cells was ~500 nt long (48, 49), whereas ATRA-induced NT2-D1 cells produced an unspliced transcript of over 4 kb as well as an ~1.1-kb spliced form (49).

Other ceRNAs arising from the *HOX* loci include *HOXA-AS2* (50, 51) and *HOTAIR* (52), which were reported to target miR-510-3p and miR-193a, respectively. In contrast to the antileukemic role of *HOTAIRM1, HOXA-AS2*, and *HOTAIR* purportedly promote AML cell survival and proliferation by freeing oncogenic pathways from the control of tumor suppressor miRNAs. *HOTAIR* was additionally reported to induce EZH2-dependent epigenetic silencing of the tumor suppressor gene *p15* (53), implying that some lncRNAs may function through multiple pathways to coordinate multilevel gene regulation. This finding is consistent with studies in other cell contexts describing how *HOTAIR* recruits polycomb repressive complex 2 (PRC2) and the LSD1/coREST/REST complex to regulate chromatin dynamics and epigenetic silencing [see Ref. (78) for a review]. On the other hand, a decisive study in human embryonic kidney 293 (HEK293) cells found that artificially tethering *HOTAIR* to chromatin led to local conformational changes and transcriptional repression independent of PRC2 (79). The matter of whether PRC2–lncRNA interactions are promiscuous or functionally specific in nature remains highly debated, with abundant evidence for both sides of the argument meriting a separate review [or three: Refs. (80–82)].

To date, there exist two other examples of lncRNAs that appear to regulate gene expression in AML by titrating miRNAs away from their endogenous mRNA targets. First, *CCAT1* was shown to inhibit myeloid maturation and promote proliferation by reducing miR-155 availability and consequently raising c-MYC levels in AML cells (54). Second, *UCA1* was reported to promote chemoresistance *via* the miR-125b/HK2 axis (57) and sustain proliferation and survival through miR-126/RAC1 (56). Of note, *UCA1* was first described in AML as a regulatory target gene of CEBPα-p30 (55)—the 30-kDa isoform that results from mutations in *CEBPA*. Hughes et al. showed that both wild-type CEBPα and the p30 protein bind the *UCA1* promoter but produce opposite effects on *UCA1* expression. Using K562 cells, they outlined *UCA1*'s oncogenic role in suppressing translation of the cell cycle regulator p27^*kip*1^ through competing for hnRNP I—a mechanism that was originally proposed in breast cancer (83) (Figure 1B). Another notable study defines *UCA1* as an RNA scaffold that is vital for normal erythrocyte development and heme biosynthesis (84).

## Other Cytoplasmic *Trans*-Regulatory Mechanisms

As illustrated by the example of *UCA1*, there is evidence of lncRNAs acting at the level of translation to regulate gene expression. In AML, the remainder of this group is represented by *PU.1-AS*, which acts on the mRNA of *PU.1*, its antisense coding gene, to reduce its translation (58). The *PU.1-AS* transcripts originate from an intronic promoter in *PU.1*, which encodes a vital transcription factor for normal hematopoiesis and whose downregulation can result in leukemogenesis [for a review, see (85)]. *PU.1-AS* was discovered to negatively regulate *PU.1* expression, through a mechanism whereby *PU.1-AS* antagonizes *PU.1* translation by selectively binding to the initiation factor eIF4A (58) (Figure 1C). *PU.1-AS* also appeared to interfere with translation elongation through an unknown mechanism. The implications of these findings on the pathophysiology of AML cells remain to be elucidated as well.

## Transcriptional/Epigenetic Regulation in the Nucleus

### In cis

**Figure 2:**
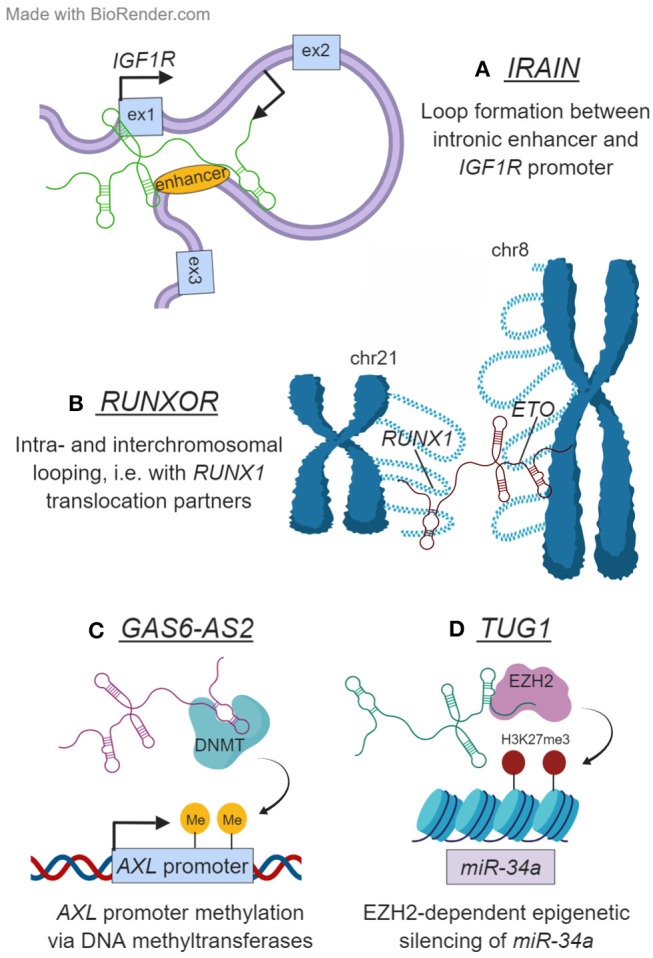
Examples of lncRNAs that function in the nucleus. **(A)** *IRAIN* is transcribed antisense to *IGF1R* and is necessary for the formation of an enhancer–promoter loop in this locus. **(B)** *RUNXOR* originates from the *RUNX1* locus on chromosome 21. Besides mediating intrachromosomal looping in this region, *RUNXOR* also contacts distant loci on other chromosomes—including the frequent *RUNX1* translocation partner *ETO* on chromosome 8. **(C)** *GAS6-AS2* modulates the expression of its head-to-head coding gene *GAS6*, as well as that of GAS6's target receptor AXL. It accomplishes the latter by depositing repressive marks on the *AXL* promoter DNA, likely with the help of a DNA methyltransferase (DNMT). **(D)** *TUG1* epigenetically regulates *miR-34a* transcription in an EZH2-dependent manner.

Besides acting on miRNAs or mRNAs in the cytoplasm, lncRNAs can also control gene expression in the nucleus at the level of transcription, epigenetics, or chromatin state. They can accomplish this *in cis* by recruiting transcription factors and epigenetic modifiers to their site of transcription or by assisting the formation of enhancer–promoter loops or other chromatin conformations. *IRAIN* is one such lncRNA. It is transcribed antisense to the gene encoding IGF1R—a key component of the PI3K/AKT signaling pathway that is constitutively active in AML—and expressed exclusively from the paternal allele in hematopoietic and leukemia cells (59). The transcript itself shows evidence of interacting with neighboring chromatin regions including the *IGF1R* promoter and an intronic enhancer (59) (Figure 2A). Indeed, Sun et al. showed that the *IRAIN* RNA is needed for an enhancer–promoter contact between these two elements. However, the impact of *IRAIN* on AML pathogenesis and whether *IRAIN*-dependent enhancer–promoter loop formation influences the expression of *IGF1R* or other genes remain to be seen. Initial hints from patient data suggest an inverse relationship between *IRAIN* and AML aggressiveness (59).

Another example of a *cis*-regulatory lncRNA is *PVT1*. The *PVT1* gene lies within the human chromosome 8q24 region, which is frequently disrupted in human cancers (i.e., by rearrangements or amplifications) and which harbors numerous *MYC* enhancer elements—including several within the *PVT1* gene locus (86). A landmark paper by Tseng et al. demonstrated *PVT1* dependence in breast cancer cells with *MYC* copy number increase (87). Consistent with the oncogenic role of *PVT1* in other cancers, its knockdown reduced proliferation and induced apoptosis in AML cell lines (60, 62, 88) and was accompanied by *MYC* downregulation in some (60, 88). A CRISPRi tiling screen covering 3 Mb of the locus identified alternative transcription start sites of two *PVT1* isoforms as enhancers that loop to the *MYC* promoter in K562 cells (61). Interestingly, in breast cancer cells, a tumor suppressor role was recently ascribed to the *PVT1* promoter, which is proposed to act as a DNA boundary element that insulates *MYC* from its downstream enhancers (89).

The *RUNX1* genetic locus on human chromosome 21 also plays a critical role in normal and malignant hematopoiesis [for a review, see Ref. (90)]. It is frequently disrupted by chromosomal aberrations in AML, such as the t(8;21) translocation that occurs in 30–40% of AML cases. In addition, the locus produces a rather unconventional lncRNA named *RUNXOR*, which is transcribed as a 216-kb unspliced sense RNA from a promoter several kilobases upstream of *RUNX1* and which, thus, overlaps the *RUNX1* introns and exons (63). Wang et al. found *RUNXOR* to be upregulated in AML patient samples compared to healthy controls, and in K562 cells following cytarabine (Ara-C) treatment. In KG-1 cells, the *RUNXOR* transcript showed evidence of interacting *via* its 3' end with the *RUNX1* promoters and enhancers (63). It also appeared to form an intrachromosomal contact with the most common *RUNX1* intronic translocation breakpoint, as well as interchromosomal contacts with distant loci including *EVI1* and *ETO*—recurrent *RUNX1* translocation partners in hematologic malignancies (Figure 2B). These latter findings suggest a revolutionary model where *RUNXOR* may physically mediate the chromosomal translocation process. Lastly, Wang et al. showed EZH2 and RUNX1 binding to the *RUNXOR* RNA, thereby implying its involvement in recruiting and directing these transcription factors (63).

### Bidirectional Promoters

The last few years have seen a steep rise in the application of large-scale CRISPR/Cas9 screens toward functionalizing lncRNAs (64, 91–98). These have been carried out in various cell systems with unified outcomes in several respects: (1) lncRNAs are indeed important in a range of cellular contexts; (2) whether they are essential or not appears to be cell type specific, sometimes even between cell lines derived from the same cancer type; and (3) the screens tend to have low hit identification rates, which may be due in part to the context-restricted manner of lncRNA expression and function.

A recent study by Bester et al. utilized a CRISPR activation (CRISPRa)-based approach to identify lncRNAs that influence cytarabine (Ara-C) resistance in AML cell lines (64). Their screen identified *GAS6*/*GAS6-AS2* as an enriched coding–noncoding gene pair in Ara-C-resistant cells. *GAS6*/*GAS6-AS2* are transcribed in opposite directions from a bidirectional promoter—a “head-to-head” configuration that qualifies *GAS6-AS2* as a divergent lncRNA. Divergent lncRNAs have been linked with the genetic loci of essential developmental transcription factors and show evidence of regulating their expression *in cis* (99–101). Consistent with this model, *GAS6-AS2* and *GAS6* expression were strongly correlated across 760 cancer cell lines, and antisense oligonucleotide-based *GAS6-AS2* knockdown also caused a significant downregulation of *GAS6* (64). In addition, AXL, the receptor of the GAS6 ligand in the TYRO3–AXL–MERTK pro-survival signaling cascade, was reduced at the mRNA level, suggesting a coexisting *trans* mechanism for *GAS6-AS2*. Based on changes in *AXL* promoter methylation upon *GAS6-AS2* knockdown and on RNA immunoprecipitation assays in K562 cells, the authors speculate that *GAS6-AS2* coordinates the activity of DNA methyltransferase proteins at the *AXL* promoter, on top of regulating *GAS6* expression (64) (Figure 2C).

### In Trans

As exemplified by *GAS6-AS2*, lncRNA transcripts can diffuse away from their genomic origin to regulate distant loci by interfacing with nuclear regulatory complexes or by acting as a scaffold for the assembly of transcriptional machinery. *TUG1*, unlike *GAS6-AS2*, seems to function exclusively *in trans*, at least so far [refer to reviews (102, 103)]. Its expression is elevated in AML patients and cell lines (65, 104) and may be linked to aurora kinase (AURKA) protein levels, leading to protection against apoptosis (104). Recent work by Li et al. moreover provides compelling evidence of *TUG1*'s role in promoting chemoresistance (65). Not only was *TUG1* upregulated in AML patients compared to healthy controls, but it was also particularly high in Adriamycin (ADR)-resistant leukemia cells relative to ADR-sensitive samples (65). In a series of complementary overexpression and knockdown experiments in HL-60 (sensitive) and HL-60/ADR (resistant) cells, Li et al. robustly demonstrated that *TUG1* confers ADR resistance through EZH2-mediated epigenetic silencing of *miR-34a* (65) (Figure 2D).

## Small RNA Host Genes

A number of miRNA host genes have been described to carry out independent regulatory functions as lncRNAs, separate from their miRNA products. Our group demonstrated this for *MONC* and *MIR100HG*, each of which harbors a three-miRNA cluster that is transcribed as a single polycistron (tricistron): *miR-99a*~*125b-2* on chromosome 21 and *miR-100*~*125b-1* on chromosome 11, respectively (66). The tricistrons are homologs of each other and were shown to protect AML blasts from transforming growth factor-β1 (TGF-β1)-mediated cell cycle arrest and apoptosis by shifting the overall balance of cellular signaling toward the Wnt pathway (105). This finding has since been corroborated by Lu et al., who showed that *MIR100HG*-derived miR-100 and miR-125b promote cetuximab resistance through the coordinated repression of five negative regulators of Wnt/β-catenin (106). They additionally discovered a double-negative feedback loop between *MIR100HG* and GATA6, in which GATA6-mediated *MIR100HG* repression is relieved through miR-125b targeting of the *GATA6* mRNA. Meanwhile, miR-125b appears to act as an oncomiR in megakaryoblastic leukemias (107). Returning to the lncRNA host genes, *MIR100HG* and *MONC* expressions are elevated in AML cell lines of the megakaryoblastic subtype (AMKL) (66) and are required for the self-renewal and maintenance of AMKL cells. When overexpressed in CD34^+^ cord blood cells, we saw that ectopic *MONC* expression interfered with hematopoietic lineage decisions and enhanced the growth of immature erythroid progenitors.

*LINC-223*, the precursor of miR-223, is a second example of a miRNA host transcript with independent lncRNA functions (67). Its expression is induced in HL-60 cells upon vitamin D3-driven monocytic differentiation, without a concomitant increase in miR-223, and similar effects were observed during monocytic differentiation of CD34^+^ cord blood progenitors (67). In order to interrogate *LINC-223* specifically, without contribution from miR-223, the authors generated a *LINC-223* derivative lacking the requisite region for DROSHA cleavage and miRNA processing. In HL-60 cells, ectopic expression of this construct impaired proliferation and cell cycle progression, meanwhile promoting monocytic differentiation (67). Mechanistically, the authors robustly demonstrated *LINC-223*'s role as a ceRNA decoy of the oncogenic miR-125 family members (miR-125a, miR-125b-1, and miR-125b-2) and implicated this lncRNA in the regulation of the transcription factor IFN4, which is typically targeted by miR-125.

## Other Regulatory lncRNAs

In this section we describe lncRNAs that show experimental evidence of regulating gene expression, but that remain ambiguous as to their precise mechanisms of action. These lncRNAs lack defined interaction partners and/or functional rescue data, making it difficult to robustly discriminate *cis* from *trans* modes of action or determine direct influence on chromatin architecture, RNA stability, translation, or regulation on any other level. Given the depth of data needed to conclusively resolve lncRNA mechanisms, many newly characterized lncRNAs (or well-known lncRNAs that are investigated in a new cell context) predictably fall in this category.

*ANRIL*, an lncRNA transcribed from the *INK4A–ARF–INK4B* cluster on chromosome 9, is known for acting through PRC1/2 to regulate the expression of *p15*^*INK*4*B*^ and *p16*^*INK*4*A*^ *in cis* and for repressing distant genes *in trans* [for a review, refer to Ref. (108)]. Polymorphisms affecting *ANRIL* have been associated with risk for various diseases including cancer, albeit with a wide range of reported impacts on the transcript itself, and *ANRIL* gain of function also gives rise to diverse cell type-dependent results despite universally increasing oncogenic phenotypes like proliferation and survival (108). This observation again highlights how lncRNAs may have context-dependent functions and discourages their generalization (108). With respect to AML, *ANRIL* was described to be essential for AML maintenance and to positively regulate glucose metabolism through ADIPOR1 and its targets, AMPK and SIRT1 (68).

The *NEAT1* lncRNA is an essential structural component of paraspeckles and plays an indispensable role in their spatial organization, possibly through long-range interactions between *NEAT1* transcripts (109). Dysregulation of *NEAT1* has been reported in human cancers: interestingly, it is upregulated in various solid tumors, whereas it is downregulated in hematologic malignancies [for recent reviews, refer to Refs. (110, 111)]. Consistent with this, *NEAT1* overexpression led to decreased proliferation and increased apoptosis in primary AML blasts and THP-1 cells (69). Zhao et al. suggested the miR-23a-3p/SMC1A axis as a downstream effector of *NEAT1* and showed that its perturbation recapitulates the *NEAT1* phenotype. Another earlier study in the APL cell line NB-4 found a subtle inverse correlation between *NEAT1* and *PML-RARA* expression and showed the upregulation of *NEAT1* upon ATRA-induced differentiation (112).

*H19* was among the first-discovered lncRNAs and has since been shown to play essential roles during development and tumorigenesis [see reviews (113, 114)]. *H19* is also quite established in the blood system, where it is one of the few lncRNAs that have been characterized in both normal and malignant hematopoiesis. The most recent data come from a single-cell resolution lncRNA landscape of murine HSC development, which identified *H19* as a critical regulator of endothelial-to-HSC transition, independent of its derivative mir-675 (115). This study further demonstrated how *H19* acts *in trans* to modulate promoter methylation of key hematopoietic transcription factors like *Runx1* and *Spi1*. In human AML cell lines, *H19* knockdown caused a decrease in proliferation and increase in apoptosis (70, 72), while elevated expression correlated with poor treatment response and overall survival in patients (72). Since its expression was observed to reciprocate that of *miR-19, H19* was proposed to act as a ceRNA upon the miR-19/ID2 axis (70). It has also been linked to telomerase activity in the NB-4 cell line, where elevated *H19* disrupted the interaction between TERT and *TR* (71). Additional studies are required to determine whether *H19* regulates both circuits at once and to resolve the mechanistic details surrounding its mode of action.

Moving this discussion to less renowned lncRNAs, *CASC15* was originally reported as a tumor suppressor in neuroblastoma (116), though it has since been found to promote oncogenic processes in melanoma (117) and other solid tumors. A study from Fernando et al. found elevated *CASC15* levels in AML and B-cell acute lymphoid leukemia (B-ALL) patients with *RUNX1* rearrangements (respectively, t(8;21)[*RUNX1-ETO*] and t(12;21)[*RUNX1-ETV6*]) compared to other cytogenetic profiles (73). Enforced expression of *Casc15* in normal murine bone marrow cells impaired their engraftment and led to a myeloid differentiation bias concurrent with defective B-cell development. The *CASC15* gene lies next to that of the B-cell transcription factor SOX4, and the pair show coordinated expression across AML and B-ALL cell lines—once again, with elevated expression in instances with *RUNX1* translocations (REH, Kasumi-1, and SKNO-1) (73). Using several strategies to deplete *CASC15* in B-ALL cell lines, Fernando et al. demonstrated its regulatory action on SOX4 and mechanistic synergy with the YY1 transcription factor. It remains unclear whether this occurs *in cis* or *in trans*, and the mechanism has yet to be validated in AML cells.

Along with *MYC* and *PVT1*, the *CCDC26* lncRNA gene lies in the human chromosome 8q21 region that is frequently amplified in cancer (86). Despite being expressed at roughly one copy per cell in myeloid leukemia cell lines, it is upregulated in these cells compared to other cancer types—including lymphoid malignancies—in which *CCDC26* expression is not detectable (74). Knockdown clones derived from K562 cells showed increased proliferation rates in serum-depleted media, as well as reduced cell death compared to control cells. This oncogenic phenotype was attributed to the induction of *KIT*, which was among the most upregulated genes in *CCDC26* knockdown cells (74).

*MEG3* is a well-known tumor suppressor lncRNA, and its expression is lost—mostly through promoter methylation—in a growing list of human cancers (118). *MEG3* was first shown to activate *p53* transcription in carcinoma cell lines (119), but a more recent study also discovered a p53-independent pathway where *MEG3* downregulates DNMT3A via MDM2/RB signaling to suppress leukemogenesis (75). The latter study further connected the transcriptional regulation of *MEG3* to WT1/TET2 (75), two frequently inactivated genes in AML. Notably, the *WT1* gene locus produces its own antisense lncRNA, *WT1-AS*, which is frequently disrupted by aberrant splicing in AML patients (120). *WT1-AS* and *WT1* expression have been linked to hypoxia-induced, TET2-dependent demethylation of the CpG island in intron 1 of *WT1* (76). *WT1-AS* additionally appears to be necessary for *WT1* expression but failed to induce *WT1 in trans* (76). Altogether, these data afford a glimpse into how complex gene regulatory networks can be steered by lncRNAs.

## Concluding Remarks

There is obviously substantial work that remains before we can claim an in-depth understanding of how lncRNAs contribute to AML biology or, indeed, to biology in general. Nevertheless, individual examples provide important insights into the regulatory roles of a few lncRNAs in the context of AML. From these, it is apparent that lncRNAs are crucial players in coordinating both novel as well as established oncogenic gene expression pathways across this heterogeneous genetic disease, whether they act upon spatial chromatin organization, epigenetics, competitive miRNA binding, or other processes. We look forward to witnessing how this new layer of regulatory complexity mediated by lncRNAs will integrate into our current protein-centric view of human health and disease and to unraveling the exquisite gene networks that will surely emerge.

## Author Contributions

MN drafted the manuscript. DH and J-HK revised the content and approved the manuscript for publication.

### Conflict of Interest Statement

The authors declare that the research was conducted in the absence of any commercial or financial relationships that could be construed as a potential conflict of interest. The reviewer SG declared a past co-authorship with the authors to the handling editor.

## Abbreviations

ADR: Adriamycin
AML: acute myeloid leukemia
AMKL: acute megakaryoblastic leukemia
APL: acute promyelocytic leukemia
Ara-C: Cytarabine
ATRA: all-trans retinoic acid
ceRNA: competing endogenous RNA
CML: chronic myeloid leukemia
CN: cytogenetically normal
CRISPR: clustered regularly interspaced short palindromic repeats
DNMT: DNA methyltransferase
*FLT3*-ITD: *FLT3* internal tandem duplications
HSC: hematopoietic stem cell
lncRNA: long noncoding RNA
LSC: leukemic stem cell
miRNA: microRNA
ML-DS: myeloid leukemia of Down syndrome
PRC: polycomb repressive complex.
